# Gut Microbiota: The Potential Key Target of TCM’s Therapeutic Effect of Treating Different Diseases Using the Same Method—UC and T2DM as Examples

**DOI:** 10.3389/fcimb.2022.855075

**Published:** 2022-03-30

**Authors:** Boxun Zhang, Ke Liu, Haoyu Yang, Zishan Jin, Qiyou Ding, Linhua Zhao

**Affiliations:** ^1^ Institute of Metabolic Diseases, Guang’anmen Hospital, China Academy of Chinese Medical Sciences, Beijing, China; ^2^ Graduate College, Beijing University of Chinese Medicine, Beijing, China

**Keywords:** gut microbiota, diabetes mellitus, ulcerative colitis, intestinal barrier, inflammation.

## Abstract

Traditional Chinese herbal medicine often exerts the therapeutic effect of “treating different diseases with the same method” in clinical practice; in other words, it is a kind of herbal medicine that can often treat two or even multiple diseases; however, the biological mechanism underlying its multi-path and multi-target pharmacological effects remains unclear. Growing evidence has demonstrated that gut microbiota dysbiosis plays a vital role in the occurrence and development of several diseases, and that the root cause of herbal medicine plays a therapeutic role in different diseases, a phenomenon potentially related to the improvement of the gut microbiota. We used local intestinal diseases, such as ulcerative colitis, and systemic diseases, such as type 2 diabetes, as examples; comprehensively searched databases, such as PubMed, Web of Science, and China National Knowledge Infrastructure; and summarized the related studies. The results indicate that multiple individual Chinese herbal medicines, such as Rhizoma coptidis (Huang Lian), Curcuma longa L (Jiang Huang), and Radix Scutellariae (Huang Qin), and Chinese medicinal compounds, such as Gegen Qinlian Decoction, Banxia Xiexin Decoction, and Shenling Baizhu Powder, potentially treat these two diseases by enriching the diversity of the gut microbiota, increasing beneficial bacteria and butyrate-producing bacteria, reducing pathogenic bacteria, improving the intestinal mucosal barrier, and inhibiting intestinal and systemic inflammation. In conclusion, this study found that a variety of traditional Chinese herbal medicines can simultaneously treat ulcerative colitis and type 2 diabetes, and the gut microbiota may be a significant target for herbal medicine as it exerts its therapeutic effect of “treating different diseases with the same method”.

## 1 Introduction

In the past decade or so, with the development of research projects such as the Human Microbiome Project and Human Intestinal Metagenome Project (Metagenomies of the Human Intestinal Tract, MetaHIT), human cognition in the field of gut microbiota has made significant progress, and the publication of relevant research results has increased exponentially (Illiano et al., 2020). Based on the evidence gathered so far, the impact of gut microbiota on health is profound and complex. Healthy gut microbiota not only prevent potentially harmful bacteria in the gut from entering the blood circulation, but also regulate metabolism and the immune system as well as provide nutrients and energy to the body. Furthermore, disorders associated with the gut microbiota are involved in the occurrence and development of various diseases, and altering this state by adjusting the diet, taking intestinal microecological agents, and even flora transplantation potentially reverse the development process of a variety of diseases to a certain extent, thus providing a novel idea for clinical treatment (Valdes et al., 2018).

Ulcerative colitis (UC) and type 2 diabetes mellitus (T2DM) are diseases closely linked to the state of the gut microbiota. As an important type of inflammatory bowel disease (IBD), UC is characterized by a continuous inflammatory response in the colorectal mucosa, which involves the rectum and damages the colon to varying degrees. Clinically, recurrent attacks and relief of alternating diarrhea, abdominal pain, mucus, pus, and blood stool are the main symptoms (Ananthakrishnan, 2015). T2DM is the main type of diabetes (accounting for > 90%), and its initial elevated blood sugar is often caused by insulin resistance. Over time, insulin production by islet beta cells gradually becomes insufficient, resulting in persistent hyperglycemia (Cho et al., 2018). Globally, the incidence of both diseases is increasing annually, and clinical evidence reveals that UC can significantly increase the risk of T2DM (Li et al., 2021c), which may be related to immune inflammation caused by intestinal flora disturbance (Jurjus et al., 2016).The combination of UC and T2DM is a difficult clinical problem. On the one hand, glucocorticoids, the first-choice treatment for UC, potentially aggravate blood-glucose fluctuation and render blood-glucose control difficult; on the other hand, hyperglycemia also makes it increasingly difficult to repair the damaged intestines of patients with UC, thus posing hidden threats to postoperative recovery (Maconi et al., 2014).

Traditional Chinese medicine (TCM) has a long history of application in China and East Asia and has significant advantages in the treatment of various complex diseases. “Treating different diseases using the same method” is an important constituent of the theoretical system of TCM; that is, when two or more diseases have the same core pathogenesis, TCM doctors may prescribe similar or exact treatments. In recent years, researchers have conducted several studies to explore the scientificity; for example, network pharmacology research has found that different diseases have related or the same pathogenesis, and TCM can play a therapeutic role in two or more diseases by regulating these core targets (Su et al., 2022). In addition, several omics studies have explored and confirmed the common characteristics of different diseases from the perspective of genes, proteins, and metabolites, and regulating these key targets is of great significance in disease treatment (Chang et al., 2018; Yang, 2019; Liu et al., 2020). Moreover, we found gut microbiota disorders to also be related to the emergence and development of a variety of diseases, and restoration of the intestinal microecology may also be a potentially important link to TCM‘s therapeutic role in “treating different diseases using the same method”.

Accumulating evidence suggests that many herbal medicines or their herbal compounds, such as *Rhizoma Coptidis* and Gegen Qinlian decoction, potentially play a therapeutic role in UC and T2DM (Zhang et al., 2013; Tian et al., 2019), and their pharmacological effects are related to the regulation of the gut microbiota. However, due to the lack of a comprehensive summary of relevant research, the utility of gut microbiota as a potential target to explain the effects of “treating different diseases with the same method” is yet to be fully elucidated. Therefore, the purpose of this review is to use UC and T2DM as examples to explain the role of the gut microbiota in the multi-pathway treatment mechanisms of TCM.

## 2 The Relationship Between UC and T2DM

### 2.1 Clinical Evidence of the Relationship Between the Two Diseases

Several studies have reported a correlation between UC onset and T2DM. A Danish, population-based cohort study found that patients with UC were significantly more likely to develop T2DM, and this risk was highest in the first year of IBD diagnosis and could persist for more than two decades (Jess et al., 2020); meanwhile, two cross-sectional studies also found the odds of suffering from T1D to increase significantly in patients with UC, a phenomenon possibly related to immune-system dysregulation (Kappelman et al., 2011; Halling et al., 2017). Moreover, a meta-analysis of five clinical studies confirmed that UC was associated with an increased risk of diabetes (odds ratio [OR]/relative risk [RR] = 1.33, 95% confidence interval [CI]: 1.03 to 1.71) (Jurjus et al., 2016). However, a retrospective cohort study from South Korea found that Crohn’s disease may increase the risk of developing diabetes; however, there was no statistical difference in patients with UC (Kang et al., 2019). In addition, UC may also promote the occurrence of other metabolic disorders besides diabetes, such as obesity, metabolic syndrome, and non-alcoholic fatty liver disease, among others (Hyun, 2021). What is the reason for this pathological phenomenon? To reveal the causes of this pathological phenomenon, researchers explored the topic from the perspective of genetics and found that there was some overlap in the susceptibility genes of patients with IBD and those of patients with metabolic diseases, such as diabetes (Lees et al., 2011).

### 2.2 Multiple Hypoglycemic Drugs Have Therapeutic Potential for UC

A retrospective study from Taiwan, China, found that patients with diabetes taking metformin had a significantly lower risk of developing UC, and a series of experimental studies found metformin to have a therapeutic effect on UC (Tseng, 2021), possibly by reducing intestinal tissue inflammation, maintaining normal mitochondrial structure, and protecting intestinal barrier integrity by regulating the TGF-β, NF-κB, LKB1/AMPK, and JNK pathways (Pandey et al., 2017; Deng et al., 2018; Samman et al., 2018; Wang et al., 2019; Liu et al., 2021). Another study found that the mucosal protective effect of metformin in UC mouse models was associated with an increased profusion of *Akkermansia muciniphila* (Ke et al., 2021). In addition, the simultaneous use of metformin and heat shock protein 90 potentially modulates HSP90/NLRP3 interaction and induces autophagy, thereby inhibiting colonic inflammation (Saber and El-Kader, 2021). Moreover, the co-administration of metformin and another hypoglycemic drug, empagliflozin, potentially exerts pharmacological effects on UC through the AMPKα/mTOR/NLRP3 signaling pathway and IL-23/IL-17 axis (Youssef et al., 2021).

In addition to metformin, other antidiabetic drugs may have potential therapeutic actions for UC. A randomized controlled clinical study confirmed that rosiglitazone, a thiazolidinedione hypoglycemic drug, was effective in the treatment of mild to moderately active UC (Lewis et al., 2008); however, a retrospective study did not support the conclusion that thiazolidinediones can treat UC (Lund et al., 2011). In addition, gliclazide, an insulin secretagogue, has also been shown to improve intestinal inflammation in UC rats *via* the PPARγ, NF-κB, and MAPK signaling pathways (Arafa et al., 2020). Several studies have also focused on the therapeutic potential of GLP-1 receptor agonists in UC, and Bang-Berthelsen et al. confirmed that GLP-1R mRNA tends to decrease by observing tissue samples from the colonic inflammation area of patients with IBD. GLP-1R-knockout IBD-model mice also showed the abnormal proliferation of microbial species in their feces and were more prone to intestinal damage, whereas the GLP-1 receptor agonist Exendin-4 and human GLP-1 analog liraglutide potentially reduced colonic inflammation through immunomodulation (Zatorski et al., 2019).

### 2.3 The Common Phenotypes Between UC and T2DM

#### 2.3.1 Gut Microbiota

A substantial body of research has provided evidence for the role of gut microbiota in the progression of UC and T2DM. The proportion of certain anti-inflammatory butyrate-producing bacteria, such as *Faecalibacterium prausnitzii* and *Roseburia* spp., decreased in the gut of patients with UC and animal models. Although some proinflammatory bacteria, such as *Fusobacterium* and *Escherichia coli*, were enriched, the diversity of the gut microbiota also demonstrated a downward trend (Pavel et al., 2021). Butyrate is a type of short-chain fatty acid (SCFA) that plays a protective role in the intestinal barrier and maintains the barrier integrity of epithelial cells by regulating the proliferation and development of intestinal epithelial cells; due to the imbalance of the microbiota structure, the total content of butyrate in the gut of patients with UC also decreases (Xu et al., 2021b). In people with T2DM, there are certain similarities in gut-microbiota changes. The abundance of some beneficial bacteria, such as *Bifidobacterium*, *Bacteroides*, *Roseburia*, *Faecalibacterium*, *and Akkermansia*, showed a downward trend (Xu et al., 2021b). However, numerous other opportunistic pathogens showed an upward trend (Qin et al., 2012). Similarly, the abundance of butyrate-producing bacteria and content of butyrate in the intestinal tract also tended to decrease in some prediabetic and T2DM populations; when this trend was reversed, the disturbance of glycometabolism also improved (Arora and Tremaroli, 2021). In addition, other intestinal metabolites, such as tryptophan and bile acids, also cause disorders in patients with UC and T2DM (Liu et al., 2022).

#### 2.3.2 Intestinal Barrier

The intestinal barrier comprises mechanical, chemical, immune, and biological barriers. Once the intestinal mucosa is damaged, bacteria and their derived toxins may infiltrate the blood circulation, enter the peripheral tissues, and induce metabolic inflammatory reactions. Intestinal-barrier damage in individuals with UC includes not only apoptosis, necrosis, and necroptosis in intestinal epithelial cells, but also disorders in tight junction protein expression, such as decreases in ZO-1 and claudin-4 and an increase in claudin-2 (Hyun, 2021). This pathological damage also occurs in individuals with T2DM. Through a retrospective analysis of capsule endoscopy data of patients with T2DM, Zhong et al. found that the incidence of intestinal villous edema and Lewis score (obtained based on intestinal villous edema, ulceration, and pathological strictures) were significantly higher than those in non-T2DM patients, and the Lewis score was positively correlated with the severity of insulin resistance (Zhong et al., 2016). Lipopolysaccharides (LPS) are important components of the cell wall of gram-negative bacteria and are released during the death and lysis of bacteria. Under physiological conditions, low concentrations of non-pathogenic LPS can be detected in circulation; however, in the state of gut-microbiota dysbiosis and intestinal mucosal damage, LPS molecules proliferate in the intestine and “leak” into the blood circulation (Cani et al., 2012). Through a prospective clinical analysis, Shen et al. found the serum LPS level in patients with T2DM to be significantly higher than that in non-T2DM patients, and LPS was positively correlated with blood-glucose fluctuation, indicating that poor blood sugar control in the short term potentially causes damage to the intestinal mucosa (Shen et al., 2019). Similarly, animal experiments further confirmed that the T2DM model also had abnormal tight junction protein expression and chronic immune inflammation in the intestinal tissue (Zhang et al., 2020); nonetheless, the degree of intestinal injury was far less than that in UC.

#### 2.3.3 Chronic Inflammation

UC pathogenesis is the result of a combination of multiple pathogenic factors, and diffuse mucosal inflammation is the main symptom (Dai et al., 2021). Recent studies have found the abnormal production of inflammatory cytokines and activation of related signaling pathways in individuals with T2DM, and gut-microbiota disorders and intestinal-barrier injury both play important roles in the pathological process of inflammation, similar to that in UC (Hotamisligil, 2006). Normally, the inflammatory response in UC is limited to the colon and rectum, while chronic inflammation in T2DM is systemic, especially in tissues or organs closely related to the pathogenesis of T2DM, such as the liver, pancreas, and fatty tissue. There are numerous overlapping inflammatory signaling pathways involved in UC and T2DM, of which the nuclear factor kappa B (NF-κB)-related pathway is representative (Jurjus et al., 2016). NF-κB plays a central role in the development of various chronic inflammatory diseases. In the resting state, NF-κB bound to the kappa-B (I-κB) inhibitor is blocked in the cytoplasm in an inactive form; however, under the influence of bacterial components (e.g., LPS), proinflammatory cytokines (e.g., TNF-α, IL-1, etc.), or viruses, the inhibitor of NF-κB kinase is activated, inducing the phosphorylation of I-κB and promoting the nuclear translocation of NF-κB (Ben-Neriah and Karin, 2011). In individuals with UC, elevated NF-κB levels in the nucleus of colonic tissue promote the release of a series of proinflammatory factors encoded by the NF-κB signaling pathway, aggravate the local inflammatory response and tissue damage, and destroy the integrity of the intestinal barrier (Lu and Zhao, 2020). In the T2DM model, increased NF-κB levels widely exist in the intestine, pancreas, and insulin-target organs (Zhang et al., 2020), and are closely related to chronic inflammation, oxidative stress, insulin resistance, and several complications (Sahukari et al., 2020). In addition, multiple other inflammatory signaling pathways may be involved in these two diseases, such as transforming growth factor-β (TGF-β), tumor necrosis factor-α (TNF-α), and peroxisome proliferator-activated receptor (PPAR), among others (Jurjus et al., 2016; Hong et al., 2018).

### 2.4 Summary

Overall, clinical studies have found a significant correlation between the occurrence of UC and T2DM, and antidiabetic drugs such as metformin can also play a significant role in the treatment of UC. In addition to genetic factors, gut-microbiota disturbance may be the “common ground” that leads to the pathogenesis of both UC and T2DM, and it not only promotes the occurrence of local inflammation in the intestinal tract, but also damages the intestinal mucosal barrier, leading to the “leakage” of LPS and the occurrence of chronic systemic inflammatory response. Thus, targeting the gut microbiota potentially exerts therapeutic effects in both UC and T2DM.

## 3 TCM Research Progress in the Treatment of UC and T2DM

To comprehensively summarize the progress of TCM research regarding the treatment of UC and T2DM simultaneously, we searched databases such as PubMed, Web of Science, and China National Knowledge Infrastructure and identified individual herbs or Chinese herbal formulations (**Tables 1**, **2**), and thoroughly excavated molecular mechanisms from the perspective of gut microbiota, intestinal barrier, and inflammatory response.

**Table 1:** Research progress of individual herb or herbal extracts for the simultaneous treatment of UC and T2DM.

Herb	Extract	Diseases	Objects	Results/Mechanisms			References
				Gut microbiota	Intestinal mucosal barrier	Inflammation	
Coptis chinensis(Huang Lian)	Berberine	UC	SD rats	**Increased** the lactic acid-producing bacteria and the carbohydrate hydrolysis bacteria **Decreased** the latent conditional pathogenic bacteria, such as *Mucispirillum, Oscillospira, B.uniformis* and *Allobaculum.*	**Decreased** ulceration, inflammatory cell infiltration, crypt damage **Restored** claudin-1 protein in colonic epithelium.	N/A	(Liao et al., 2020)
	Berberine	UC	Balb/c	N/A	**Increased** expression levels of Claudin-1, Occludin and ZO-1 in colonic tissue	**Decreased** TNF-α, IL-1β**Increased** IL-10 levels in colonic tissues	(Shen et al., 2017; Shen et al., 2018)
	Berberine	UC	Human	**Increased** Chao index, ACE index, Shannon index of the gut microbiota	N/A	**Decreased** IL-1β, IL-6, IL-8, TNF-α levels in the serum	(Gan et al., 2020)
	Oxyberberine	UC	Balb/c	**Restore** the overall structure of the gut microbiota **Decreased** the abundance of Firmicutes and Clostridium, Coprobacillus, Dehalobacterium, Parabacteroides, Paraprevotella, Ruminococcus, Staphylococcus	**Enhanced** the expressions of mucin mRNA (mucin-1, mucin-2) and TJs proteins(ZO-1,ZO-2,Occludin,JAM-A,Claudin-1)	**Decreased** colonic inflammatory cytokines TNF-α, IL-1β, IL-6, IL-17, IFN-γ and IL-10 **Inhibited** TLR4-MyD88-NF-κB signaling pathway	(Li et al., 2020)
	Dihydroberberine	UC	Balb/c	N/A	**Enhanced** the expressions of mucin mRNA (mucin-1, mucin-2) and TJs proteins(ZO-1,ZO-2,Occludin,JAM-A,Claudin-1)	**Decreased** colonic inflammatory cytokines TNF-α, IL-1β, IL-6, IL-17 and IFN-γ Blocked TLR4/MyD88/NF-κB signal pathway of the colon	(Li et al., 2021)
	Crude Coptis Polysaccharide+Berberine	UC	Balb/c	N/A	**Increased** expression levels of Claudin-1, Occludin and ZO-1 in colonic tissue	**Improved** the pathological manifestation of inflammatory cell infiltration in intestinal recess	(Xue et al., 2022)
	Water extract	UC	SD rats	**Increased the abundance of probiotics such as** *Akkermansia* and *Blautia* Decrease **the abundance of** pathogenic bacteria such as *Escherichia-Shigella* and *Clostridium_sensu_stricto_1*	**Increased** protein expression levels of Occludin and ZO-1, and the mRNA expression of Claudin-5 in colonic tissue	**Decreased the** expression of TNF-α, COX-2 and iNOS	(Xie et al., 2022b)
	Berberine	T2DM	SD rats	Increased the community richness and diversity of the gut microbiota Increased the abundance of Bacteroidetes and Lactobacillaceae Decreased the of abundance of Proteobacteria and Verrucomicrobia, and reduced intestinal metabolites tyrosine, tryptophan and phenylalanine	N/A	N/A	(Yao et al., 2020)
	Berberine	T2DM	SD rats	N/A	**Restored** the tight junction protein ZO-1 **Increased** villi/mucosa height **Ameliorated** detachment of villi/epithelial	**Decreased** infiltration of inflammatory cells plasma and LPS level in plasma	(Shan et al., 2013)
	Berberine	T2DM	db/db mice	**Increased** the abundance of SCFA-producing bacteria. **Reduced** the abundance of gut microbiota **Decreased** the abundance of opportunistic pathogens. **Recovered** the total SCFA content.	**increased** expression of both Occludin and ZO-1 **Narrowing** the intestinal epithelial intercellula gaps.	**Decreased** serum LPS level. **Down-regulated** of intestinal TLR/NF-κB signaling activities.	(Zhang et al., 2019)
Rhizoma curcumae longae (Jiang Huang)	Curcumin	UC	Balb/c mice	**Improved** the gut microbiota diversity. **Increased** *Brevibacterium* and *Sphingobacterium*. **Decreased** the relative abundance of *Sporosarcina*, *Alloprevotella*, *Ruminiclostridium*, *Pevotellaceae_UCG-001*, and *norank_f_Ruminococcaceae.*	**Decreased** the inflammatory cell infiltration. **Promoted** the mucosal integrity	**Inhibited** the expression of proinflammatory cytokines IL-1β, IL-2, IL-6, IL-9, and IL-17A and the activation of PI3K/Akt/Raptor/Rictor signaling pathway in colonic tissue.	(Zhong et al., 2021)
	Curcumin	UC	SD rats	**Increase** the contents of *Bifidobacterium* and *Lactobacillus.* **Decrease** the contents of *Escherichia coli* and *Enterococcus.*	**Decreased** Inflammatory cell infiltration in colonic tissues **Promoted** the Mucosal integrity and the healing of ulcers.	**Increased** the anti-inflammatory cytokine IL-4 levels in serum and the expression of NF-κВ colonic tissue. **Decreased** IL-1β and IL-6 levels, TNF-α and IL-8 expression in serum.	(Huang et al., 2017)
	Curcumin	Chronic inflammatory disease	Intestinal epithelial cells(Caco-2 and HT-29) and macrophages	N/A	**Reduced** MLCK expression **Prevented** LPS-induced disorganization of ZO-1, Claudin-1, Claudin-7 as well as actin filaments	**Decreased** the secretion of IL-1β and the phosphorylation/activation of p38 MAPK **Increased** the secretion of IL-10.	(Wang et al., 2017)
	Curcumin	T2DM	SPF rats	**Increased** *Bacteroidetes* and *Bifidobacterium* **Suppressed** *Enterobacterales* and *Firmicutes*	**Decreased** the contents of DAO in the serum. **Promoted** the expression of ZO-1 and occluding in in the ileal tissue.	**Decreased** LPS, TNF-α, and TLR4/NF-κB signaling pathway in the serum.	(Huang et al., 2021)
	Tetrahydrocurcumin	T2DM	db/db mice	**Decreased** the relative abundance of *Proteobacteria*, *Actinobacteria* and the ratio of Firmicutes to Bacteroidetes.	N/A	N/A	(Yuan et al., 2020a)
Radix Astragali seu Hedysari (Huang Qi)	Astragalin	UC	C57BL/6J mice	**Increased** the abundance of potentially beneficial bacteria such as *Ruminococcaceae*. **Decreased** the abundance of potentially harmful bacteria such as *Escherichia-Shigella*.	**Increased** ZO-1, occludin, and Muc2 in colonic tissue. **Improved** intestinal mucosal barrier function.	**Down-regulated** TNF-α, IL-6, IL-1β levels and the NF-κB signaling pathway in the colon. **Inhibited** colonic infiltration by macrophages and neutrophils, ameliorated metabolic endotoxemia.	(Peng et al., 2020)
	Astragaloside IV	T2DM	Kunming mice	**Increased** *Anaerobacter, Romboutsia*, *Alkalibacteria, Canadidatus stoquefichus, Oligobacterium, Brautella, Erysipelatoclostridum.* **Decreased** *Bacteroides, Oscillibacter, Parabacteroides, Roseburia Muribaculum.* **increased** butyric acid levels.	**Decreased** the permeability of the intestinal mucosa.	**Up-regulated** AMPK/Sirt1 and PI3K/AKT signaling pathways in liver tissues.	(Gong et al., 2021)
Radix Ginseng (Ren Shen)	Ginsenoside Rg1	UC	Balb/c mice	**Increased** *Lachnospiraceae.* **Decreased** *Staphylococcus, Bacteroide and Ruminococcaceae_UCG_014.*	**Inhibited** the pathological damage to the colon. **Decreased** ulcer formation and inflammatory cell infiltration.	**Down-regulated** IL-6, IL-33, CCL-2 and TNF-α; **Up-regulated** IL-4 and IL-10in colonic tissues. **Inhibits** the activation of Nogo-B/RhoA signalling pathway.	(Long et al., 2022)
	Ginsenoside Rg5	T2DM	Lepr^db^ mutant db/db mice	**Increased** *Bacteroides, Alloprevotella, Parabacteroides, Ruminococcus, Clostridium XVIII, Butyricicoccus, Vampirovibrio.* **Reduced** *Lactobacillus, Akkermansia, Acetobacteroides, Acetatifactor, Enterorhabdus, Anaerotruncus, Intestinimonas.*	**Increased** the expression levels of occludin and ZO-1.	**Reduced** the protein expression of TNF-α, IL-6, IL-1β in serum and in liver tissues. **Dcreased** LPS levels. **Inhibited** TLR4-related inflammatory signaling pathways.	(Wei et al., 2020)
Rhizoma Atractylodis Macrocephalae(Bai Zhu)	Polysaccharides	UC	C57BL/6J mice	**Increased** community richness and reversed the disturbed structure of the gut microbiota. **Increased** *Lactobacillus* and *Butyricicoccus*.	**Rescued** the destructed tissue architecture, as evidenced by restoration of crypt structure and reduction of inflammatory cell infiltration. **Reduced** histopathological score.	**Decreased** proinflammatory cytokines including TNF-a, IL-18, IL-1β in the colon.	(Feng et al., 2020)
	Ethanol extract	T2DM	db/db mice	**Increased** *B. thetaiotaomicron* and *M. smithii*	**Attenuated** intestinal damage including the improvement of disorganized and collapsed intestinal villi, and swollen and degenerated villus epithelium.	**Decreased** IL-1β Levels and LPS in serum. **Regulated** the protein expression of NF-κB p65 and PI3K/FOXO1/PDX-1 signaling pathways in hepatic and pancreatic tissues.	(Zhang et al., 2017)
Folium Mori (Sang Ye)	Albumin and enzymatic hydrolysates	UC	C57BL/6 mice	**Increased** α diversity of gut microbiota and the abundance of *Muribaculaceaek*. **Decreased** Firmicutes/Bacteroidetes ratio and the abundance of *Lachnospiraceae*. **Increased** the contents of acetic acid, propanoic acid, and butyric acid.	**Improved** the integrity of the colon and alleviates the goblet cell reduction and mucin secretion caused by colitis	**Inhibited** the production and gene expression of pro-inflammatory cytokines (IL-6, TNF-α, IL-1β) in the colon.	(Tang, 2020)
	Herbal powder	T2DM	SD rats	**Increased** *Phascolarctobacterium*, *Ruminococcus*, *Oscillospira*, *Ruminococcaceae*, *Ruminococcus*, Clostridium and S24-7, *Prevotella*, *Parabacteroides*, *Prevotella*, *Bacteroides*. **Decreased** *Bifidobacterium*, *Collinsella*, *Eubacterium*, *Coprobacillus* and *Dorea*.	N/A	N/A	(Sheng et al., 2017)
	Herbal extract	T2DM	Male ICR mice	N/A	N/A	**Reduced** TNF-α in the serum. **Suppressed** TLR2 signalling pathway, stimulating the insulin signalling pathway and the interactions of the two through TNF-α.	(Tian et al., 2019)
Radix Salviae Miltiorrhizae (Dan Shen)	Salvianolic Acid A	UC	SD rats	**Increased** *Firmicute*, *Verrucomicrobia Akkermansia*, *Bacillus*, *Blautia*, *Lachnoclostridium*, and *Lactobacillus*. **Decreased** the relative abundance of the *Bacteroides*, *Roseburia*, and *Ruminiclostridium*.	**Increased** the mRNA expression of occludin and ZO-1 in colonic tissue.	**Increased** the anti-inflammatory cytokine TGF-β in the colonic mucosa. **Decreased** the pro-inflammatory cytokines (IL-1β, MCP-1, and IL-6) in the colonic mucosa.	(Wang et al., 2018a)
	Herbal extract	T2DM	C57BL/6J mice	**Increased** the diversity of gut microbiota and the relative abundances of *Deferribacteres, Anaerotruncus colihominis, Mucispirillum schaedleri, Butyricimonas virosa.* **Decreased** *Firmicutes/Bacteroidetes* ratio and the relative abundances of *Proteus hauseri, Helicobacter winghamensis.*	**Up-regulating** the tight junction proteins expressions (ZO-1, Occludin and Claudin-5) in ileum and colon. **Reversed** the abnormal VIP and AGEs level in DM mice to lighten intestinal damage.	N/A	(Gu et al., 2017)
Radix Scutellariae (Huang Qin)	Polysaccharide	UC	C57BL/6 mice	**Increased** the abundance of Firmicutes, *Bifidobacterium*, *Lactobacillus*, and *Roseburia*. **Inhibit** the levels of *Bacteroides*, *Proteobacteria* and *Staphylococcus*. **Restored** the contents of acetic acid, propanoic acid, and butyric acid.	**Decreased** histological score of colonic tissue **Up-regulated** expressions of ZO-1, Occludin and Claudin-5.	**Decreased** the protein expressions of IL-6, IL-1β, and TNF-α in colon tissues and serum. **Reduced** blood LPS levels.	(Cui et al., 2021)
	Water extract	T2DM	SD rats	**Increased** *Blautia, Lachnoclostridium, Ruminiclostridium_5, Turicibacter*. **Decreased** the relative abundances of *Lactobacillus* and *feacalibaculum*. **Restored** the contents of secondary BAs in the serum or faeces.	N/A	N/A	(Zhao et al., 2021)
	Water extract	T2DM	SD rats	**Increased** *Bacteroidales S24-7group_norank*, *[Eubacterium] nodatum group Parasutterella*, *Prevotellaceae UCG-001*, *Ruminiclostridium*, *Ruminiclostridium 9.* **Increased** Acetic acid, Propionic acid, Butyric acid, Isobutyric acid,Valeric acid, Isovaleric acid. **Decreased** *Escherichia-shigella.* and Firmicutes to Bacteroidetes ratio	**Alleviated** infiltration of inflammatory cells including monocytes and neutrophils.	**Decreased** proinflammatory cytokines TNF-α, IFN-γ, IL-1β in serum.	(Xiao et al., 2020)
Rheum officinale (Da Huang)	Rhein	UC	C57BL/6J mice	**Altered** gut microbiota composition **Increased** *Lactobacillus*.	**Decreased** immune cell infiltration and tissue damage **Restored** claudin-1, E-cadherin expression, and mucus secretion.	**Decreased** IL-17, CXCL1, and IFN-γ in colon. **Down-regulated** the IL-17/IL-10 ratio in colon.	(Wu et al., 2020)
	Rhein	T2DM	C57BL/KsJ-db/db mice	**Increased** the relative abundance of *Bacteroides* and *Akkermansia*. **Decreased** the ratio of Bacteroidetes and Firmicutes.	**Increase** the number of L-cells in the terminal ileum.	N/A	(Wang et al., 2018b)
	Rhubarb Root Extract	prediabetes	C57BL/6J mice	**Increased** the abundance of *Akkermansia muciniphila*, *Parabacteroides* and *Erysipelatoclostridium* **Decreased** the abundance of *Ruminococcus* and *Peptococcus*	**Increased** the mRNA expression of antimicrobial peptides Reg3g and Pla2g2 **Increased** the mRNA expression of Intectin(a key protein involved in intestinal epithelial cell turnover)	**Decreased** hepatic inflammation markers RANTES, TNF-α, IL-6 and IFN-γ **Decreased** the mRNA expression of inflammatory markers TNF, IL-10, Lbp and Itgax in the adipose tissue	(Régnier et al., 2020)
	Alcohol extract	T2DM	SD rats	**Increased** the abundance of probiotic *Lactobacillus* and SCFA-producing bacteria **Decreased** the abundance of the *Lachnospiraceae NK4A136 group* and LPS-producing *Desulfovibrio*	**Increased** the expression level of ZO-1 and occludin in the ileum tissues	N/A	(Cui et al., 2019)
Centella asiatica (Ji Xuecao)	Ethanol extract	UC	Balb/c mice	**Rehabilitated** the structure and constitute of integral microbiota community. **Decreased** *Firmicutes, Proteobacteria,Helicobacter, Jeotgalicoccus, Staphylococcus.*	**Promoted** the expressions of ZO-1, E-cadherin **Protecting** the colonic mucosal epithelial structure	N/A	(Li et al., 2021a)
	Ethanol extract	T2DM	ZDF rats	**Regulated** the relative abundances of *Bacteroidetes* and *Firmicutes.* **Decreased** the relative abundances of *Prevotellaceae.*	N/A	**Decreased** serum levels of TNF-α.	(Si, 2016)
Herba Dendrobii (Shi Hu)	Polysaccharide	UC	C57BL/6 mice	**Increased** *Romboutsia, Lactobacillus* and *Odoribacter*. **Decreased** *Parasutterella, Burkholderia-Caballeronia-Paraburkholderia, Acinetobacter.*	**Increased** the protein and mRNA expression levels of Occludin and ZO-1. **Decreased** endotoxin, DAO and D-lactic acid in serum and MPO content in colon tissue.	**Increased** IL-5, IL-10, IL-22, IFN-γ, TNF-α and TGFβ1 in the colon. **Decreased** IL-1β, IL-6, IL-17A, IL-17F, IL-21 and IL-23 in the colon. **regulated** the Nrf2/NF-κB signaling pathways.	(Wang et al., 2022)
	Rich-Polyphenols Extract	T2DM	db/db mice	**Increased** *Bacteroidetes* to *Firmicutes* ratios, *Prevotella, Akkermansia* **Decreased** *S24-7, Rikenella, Escherichia coli.*	N/A	**Decreased** IL-6 in serum and TNF-α in liver/kidney tissue.	(Li et al., 2018)
Licorice (Gan Cao)	Licoflavone B	UC	C57BL/6	**Increased** the relative abundances of beneficial microorganisms (*Bacteroides* et al.) **Decreased** the Firmicutes/Bacteroidetes ratio and the relative abundances of harmful bacteria (*Enterococcus* et al.)	**Inhibited** colonic cell apoptosis **Protected** the expression of occludin, claudin-1, and ZO-1	**Inhibited** the expression of MAPK pathway-related proteins ERK, p38 and JNK	(Zhang et al., 2022)
	licorice extract	T2DM	Kunming mice	**Increased** the contents of *Alloprevotella*, *Bacteroides*, and *Akkermansia* **Decreased** the contents of *Lachnospiraceae_NK4A136_group*	**Improved** the pathological change of colon tissue in diabetic mice, such as the significant inflammatory cell infiltration, the loss of goblet cells, and the branching and shrunk crypts	**Regulated** the colon TLR4/NF-κB signaling pathway(decrease the levels of TLR4, NF-κB, IKK-α, IκBα and TNF-α)	(Zhang et al., 2022b)

UC, ulcerative colitis; T2DM, type 2 diabetes mellitus; N/A,not applicable; TNF-α:tumor necrosis factor-α; IL,interleukin; ZO-1,zonula Occludens; JAM-A, junctional adhesion molecule-A; IFN-γ: interferon-γ; TLR4, toll-Like Receptor 4; MyD88, myeloid differentiation factor 88; NF-κB, nuclear factor of kappa B; COX-2, cyclooxygenase 2; iNOS, nitric oxide synthase; SCFA, short chain fatty acid; LPS, lipopolysaccharide; PI3K, phosphoinositide 3-kinase; MLCK, myosin light chain kinase; MAPK, mitogen-activated protein kinase; DAO, diamine oxidase; MUC2, mucin 2; AMPK, AMP-activated protein kinase; Sirt1, silent information regulator 1; CCL-2, chemokine CC ligand-2; Nogo-B, Reticulon 4B; FOXO1, forkhead box class O1; PDX1, pancreatic and duodenal homeobox gene-1; TGF-β: transforming growth factor β; MCP-1, monocyte chemoattractant protein-1; AGE, advanced glycation end products; BAs, bile acids; CXCL1, Chemokine (C-X-C motif) Ligand 1; Reg3g, regenerating islet-derived protein 3 gamma; PLA2G2A, phospholipase A2 group IIA; LBP, lipopolysaccharide-binding protein; Itgax, tegrin alpha X; TGFβ1, transforming growth factor beta1; Nrf2, nuclear factor E2 related factor 2; ERK, extracellular signal regulated kinase; JNK, C-Jun N-terminal kinase; IKKα: inhibitor of kappa B (IκB) kinase α; IκB, inhibitor of NF-κB.

### 3.1 Individual Herbs or Herbal Extracts

Berberine and curcumin are well-known herbal extracts whose hypoglycemic effects have been confirmed by multiple clinical studies (de Melo et al., 2018; Liang et al., 2019); moreover, these two extracts have also proven to improve intestinal inflammation (Zhang et al., 2016; Li et al., 2020). Berberine is an active alkaloid isolated from *Rhizoma coptidis*, and the regulation of the intestinal microbiota is a key target for berberine’s multiple pharmacological effects (Habtemariam, 2020; Cheng et al., 2021). In animal models of UC and T2DM, berberine increased the number of beneficial bacteria and reduced the potential pathogenic bacteria, thereby reducing inflammatory cell infiltration, repairing the intestinal mucosal barrier, and playing a role in the treatment of UC (Liao et al., 2020). In addition, it potentially reduces serum LPS levels, thus alleviating the chronic inflammatory state by downregulating the TLR/NF-κB signaling pathway (Zhang et al., 2019). Curcumin, a polyphenol extracted from *Rhizoma curcumae longae*, can repair dysregulated gut microbiota and exert various pharmacological effects. On the one hand, it can regulate the balance of Treg/Th17 cells in patients with UC and inhibit the expression of inflammatory factors, such as IL-6 and IL-17A (Zhong et al., 2021); on the other hand, it also prevents the LPS “leakage,” inhibits the occurrence of systemic inflammatory responses, and protects pancreatic islets and insulin target organs (Huang et al., 2021).

In the TCM theory system, *Radix astragali seu Hedysari*, *Rhizoma atractylodis macrocephalae*, and *Radix ginseng* are considered to have the effect of “invigorating qi” and potentially treat various debilitating diseases. *Radix astragali seu Hedysari* is a perennial Compositae plant, and its extract has been shown to increase butyrate-producing bacteria in the intestine, modulate the PI3K signaling pathway in T2DM mice (Gong et al., 2021), and repair the damaged intestinal mucosal barrier in mice with UC by reducing the pathogenic bacteria *Escherichia* spp. and *Shigella* spp. (Peng et al., 2020). *Rhizoma atractylodis macrocephalae* is often used as a beneficial spleen herb in China because of its unique advantages in improving gastrointestinal dysfunction (Yang et al., 2021a). Polysaccharides are important active ingredients of *Atractylodes macrocephala*, and Feng et al. found *Atractylodes* polysaccharides to increase beneficial bacteria, such as *Lactobacillus* and *Butyricicoccus*, affect the metabolic process of amino acids and bile acids, and reverse the damaged intestinal-tissue structure in UC model mice (Feng et al., 2020); in addition, its ethanol extract can also repair intestinal mucosal injury and reduce serum IL-1β and LPS levels in T2D mice (Zhang et al., 2017). Saponins are the main active components of ginseng and have anti-inflammatory and metabolic regulatory effects (Gao et al., 2020). In mouse models of UC and T2D, ginsenosides regulate gut microbiota, thereby improving intestinal mucosal damage and a series of inflammatory reactions (Wei et al., 2020; Long et al., 2022).

Furthermore, some herbs, such as *Folium mori*, *Radix salviae miltiorrhizae*, and *Radix scutellariae*, among others, also have dual therapeutic effects on UC and T2D, and more details are summarized in **Table 1**.

### 3.2 Chinese Herbal Formulae

Chinese herbal formulae are an ingenious combination of several herbs under the guidance of TCM theory, which are characterized by multicomponent, multitarget, and synergistic effects (Qiu, 2007). Gegen Qinlian decoction (GGQLD), Huanglian Jiedu decoction (HLJDD), and Huangqin decoction (HQD) are considered to have the “clearing heat” effect, and modern research suggests that they have obvious anti-inflammatory effects and could be widely used in acute or chronic inflammatory diseases (Gao et al., 2017). GGQLD was first recorded in the book Treatise on Febrile and Miscellaneous Diseases written by Zhongjing Zhang, mainly comprising Gegen (*Radix puerariae*), Huangqin (*Radix scutellariae*), Huanglian (*Rhizoma coptidis*), and Gancao (*Radix glycyrrhizae*). The effects of GGQLD on the treatment of UC and T2DM are closely related to gut-microbiota regulation, and in the dextran sulfate sodium (DSS)-induced UC rat model, it potentially increased the relative abundance of the *Lachnospiraceae_NK4A136_group* and *Roseburia* (Li et al., 2021); in a T2DM rat model, it potentially increased butyric acid-producing bacteria, such as *Faecalibacterium*, *Roseburia*, *Clostridium XIVa*, and beneficial bacteria, such as *Flavonifractor* and *Acetatifactor* (Xu et al., 2020; Tian et al., 2021). In addition, GGQLD may also increase the expression of ZO-1 and occludin and downregulate inflammatory factors IL-6, IL-1β, TNF-α, IFN-γ, and IL-17 in both disease models, thereby repairing the intestinal mucosal barrier and reducing the level of inflammation in the intestine and circulation. Additionally, Shenling Baizhu Powder (SLBZP) and Lizhong Decoction (LZD) are considered to have the effect of “invigorating the spleen and regulating the stomach”, while Banxia Xiexin Decoction and Wumei Decoction are the representative formulae for treating diseases characterized by the cold-heat complex. In modern clinical practice, the above formulae have been widely used in various gastrointestinal and metabolic diseases, and the gut microbiota may be a potential key target. More details are listed in **Table 2**.

**Table 2:** Research progress of herbal formulae for the simultaneous treatment of UC and T2DM.

Herbal formulae	Diseases	Objects	Results/Mechanisms			References
			Gut microbiota	Intestinal mucosal barrier	Inflammation	
Gegen Qinlian Decoction	UC	SD rats	**Increased** the microbial α-diversity and the abundance of *Blautia, Akkermansia, Erysipelatoclostridium*, *Roseburia* and so on. **Increased** the contents of butyrate, propionate, hexanoate, isohexanoate and total-SCFAs. **Decreased** the community abundance of *Bacteroides, Escherichia-Shigella, Allobaculum, [Ruminococcus]_gauvreauii_group and Romboutsia.*	**Decreased** the activities of DAO and D-lactate in serum **Increase** the expressions of ZO-1 and Occludin.	**Down-regulated** inflammatory factors IL-6, IL-1β, TNF-α, IFN-γ, IL-17 and the levels of MIP-1α, MIP-1β and CXCL1 in colon. **Up-regulate** the level of IL-10 in colon.	(Li et al., 2021b)
	T2DM	GK rats	**Enriched** *Faecalibacterium, Roseburia, Clostridium XIVa, Ruminococcus2, Dorea, Parabacteroides, Paraprevotella, Butyricimonas, Alistipes, Gemmiger, Butyricicoccus*, and *Coprococcus.*	N/A	**Reduced** IL-6, IL-17, TNF-a, IFN-γ, MCP-1, IL-1β in the serum. **Down-regulated** the expression levels of NF-κB1 and Stat1.	(Xu et al., 2020)
	T2DM	Wistar rats	**Increased** beneficial bacteria such as *Flavonifractor* and *Acetatifactor.* **Decreased** opportunistic pathogens such as *Butyricimonas, Anaerofustis, Butyricicoccus, Gammaproteobacteria.*	**Increased** the expression levels of ZO-1, Claudin-1, and Occludin.	**Reduced** the expression levels of CRP, IL-1β, TNF-α, MCP-1, and endotoxins in the serum.	(Tian et al., 2021)
	T2DM	Human	**Increased** *Faecalibacterium, F*. *prausnitzii*, *Bifidobacterium*, *Gemmiger.* **Decreased** *Alistipes*, *doribacter*.	N/A	N/A	(Xu et al., 2015)
Huanglian Jiedu Decoction	UC	Balb/c mice	**Increased** the relative abundance of *Oscillibacter, Lactobacillus, Clostridium_IV, Desulfovibrio, Clostridium_XIVa.* **Decreased** the α diversity of the intestinal flora and the abundance of *Parabacteroides*.	**Improved** damaged colonic mucosal epithelium. **Decreased** inflammatory cells infiltrated.	**Decreased** the levels of IL-1β and TNF-α in plasma. **Increased** the level of IL-10 in plasma.	(Yuan et al., 2020b)
	UC	Balb/c mice	N/A	**Increased** the secretion of mucin and the expression of ZO-1 and occludin in colonic mucosa	**Up-regulated** plasma IL-10, down-regulated TNF-α and IL-1β levels **Inhibited** the expression of NF-κB p65, p-IκKα/β, and p-IκBα proteins in the colon	(Yuan et al., 2019)
	T2DM	SD rats	**Increased** *Parabacteroides, Blautia*, and *Akkermansia.* **Decrease** *Aerococcus, Staphylococcus, Corynebacterium.* **Up-regulation** in bile acid biosynthesis.	N/A	**Decreased** inflammatory factors IL-1β, CRP, MDA in serum.	(Chen et al., 2018)
Huangqin decoction	UC	C57BL/6 mice	**Increased** *Lactococcus.* **Decreased** *Desulfovibrio, Helicobacter.*	**Protected** colon crypt structures. **Decreased** histologic inflammation.	**Suppressed** TNF-α, IL-6, IL-1β, and COX-2 in colorectum.	(Yang et al., 2017)
	UC	Balb/c mice	**Recovered** the gut microbiota diversity **Decreased** the ratio of Firmicutes/Bacteroidetes and the Abundance of Proteobacteria	**Inhibited** the MPO activity of colon tissue **Prevented** neutrophil infiltration to protect the intestinal epithelial cell barrier	**Decreased** the colonic level of LPS. **Decreased** the mRNA levels of TLR-4, IL-1β, IL-6, IL-4 and IL-10 in colon **Inhibited** colonic related expression levels of PI3K-AKT-HIF-1a and NF-κB pathways	(Li et al., 2019a)
	T2DM	Human	**Increased** Bacteroidetes and Bacteroidaceae.	N/A	N/A	(Cao et al., 2019)
Banxia Xiexin Decoction	UC	C57BL/6J mice	**Increased** *Dubosiella, Bacteroides, Allobaculum, Bifidobacterium.* **Decreased** the ratio of *Firmicutes/Bacteroidota* and the abundance of *Lactobacillus, Clostridium_sensu_stricto_1, Enterorhabdus, Candidatus_Saccharimonas, Eubacterium_fissicatena_group.*	**Reduced** inflammatory cell infiltration of colon mucosa. **Improved** the integrity of epithelial cells.	**Reduced** IL-6 and TNF-α in serum.	(Chen et al., 2021)
	T2DM	SD rats	**Increased** *Intestinibacter, Sphingomonas, Enterococcus, Ruminococcaceae UCG-014*. **Increased** levels of acetic, butyric, pentanoic and hexanoic acids.	**Reduced** inflammatory cell infiltration. **improved** ileum morphology	N/A	(Yang et al., 2021b)
Shenling Baizhu Powder	UC	Human	**Increased** *bifidobacterium,lactobacillus.* **Decreased** *enterobacter, enterococcus, clostridium, bacteroides.*	N/A	N/A	(Yang et al., 2021)
	T2DM	ZDF rats	**Decreased** *Acinetobacter, Lactobacillus, Roseburia, Staphylococcusc.* **increased** *Anaerostipes, Turicibacter, Bilophila, Ochrobactrum, Psychrobacter, Prevotella.*	N/A	**Decreased** IL-1β, MCP-1 in serum.	(Zhang et al., 2021)
Lizhong Decoction	UC	C57BL/6 mice	**Increased** *Bacteroidetes*, *Blautia*, Muribaculaceae_norank, Prevotellaceae UCG-001, Ruminiclostridium 9. **Decreased** the ratio of *Firmicutes* to *Bacteroidetes*, *Proteobacteria, Clostridiumsensu stricto1, Enterobacter, Escherichia-Shigella.*	N/A	N/A	(Zou et al., 2020)
	UC	C57BL/6 mice	N/A	**Increased** the expression levels of ZO-1, occludin and claudin-1	**Decreased** TNF-α, IFN-γ, IL-6, IL-8 and IL-1β in the colon. **Increased** IL-10,IL-4 in the colon and the levels of NF-κB and TLR4.	(Shen et al., 2020)
	T2DM	human	**Increased** *Bifidobacterium.* **Decreased** *Enterococcus faecalis.*	N/A	**Decreased** MCP-1, TNF-α, hs- CRP in serum. **Increased** IL-10 in serum.	(Chen et al., 2020)
Wumei Decoction	UC	C57BL/6 mice	**Increased** *Allobaculum* and *Bacteroides*. **Decreased** *Ileibacterium*.	**Promoted** the expression of claudin-4, claudin-5, claudin-8, E-cadherin, occluding, ZO-1. **Down-regulated** the expression of claudin-1.	**Decreased** TNF-α, IFN-γ, and IL-17A in colon.	(Wu et al., 2022)
	T2DM	SD rats	**Increased** *Firmicutes, DeltaProteobacteria, Lactobacillus*. **Decreased** *Bacteroidetes, Actinobacteria, Bacteroides, Clostridium*. **Increased** the contents of acetic acid, propionic acid and n-butyric acid.	N/A	**Decreased** TNF-α in serum. **Increased** IL-10 in serum.	(Zhou et al., 2020)

UC, ulcerative colitis; T2DM, type 2 diabetes mellitus; N/A,not applicable; SCFA, short chain fatty acid; DAO, diamine oxidase; ZO-1,zonula Occludens-1; IL,interleukin; TNF-α:tumor necrosis factor-α; IFN-γ: interferon-γ;MIP, macrophage inflammatory protein; CXCL1, Chemokine (C-X-C motif) Ligand 1; MCP, monocyte chemokine-1; NF-κB, nuclear factor of kappa B; Stat 1, signal transducer and activator of transcription 1; CRP, C-reactive protein; IκKα/β: IκB kinase α/β; IκB, inhibitor of NF-κB; MDA, malondialdehyde; COX-2, cyclooxygenase 2; MPO, myeloperoxidase; PI3K, phosphoinositide 3-kinase; HIF-1, hypoxia inducible factor-1.

## 4 Discussion

### 4.1 Gut Microbiota: A Potential Key Target of Herbal Medicine in the Simultaneous Treatment of UC and T2DM

**Figure 1:**
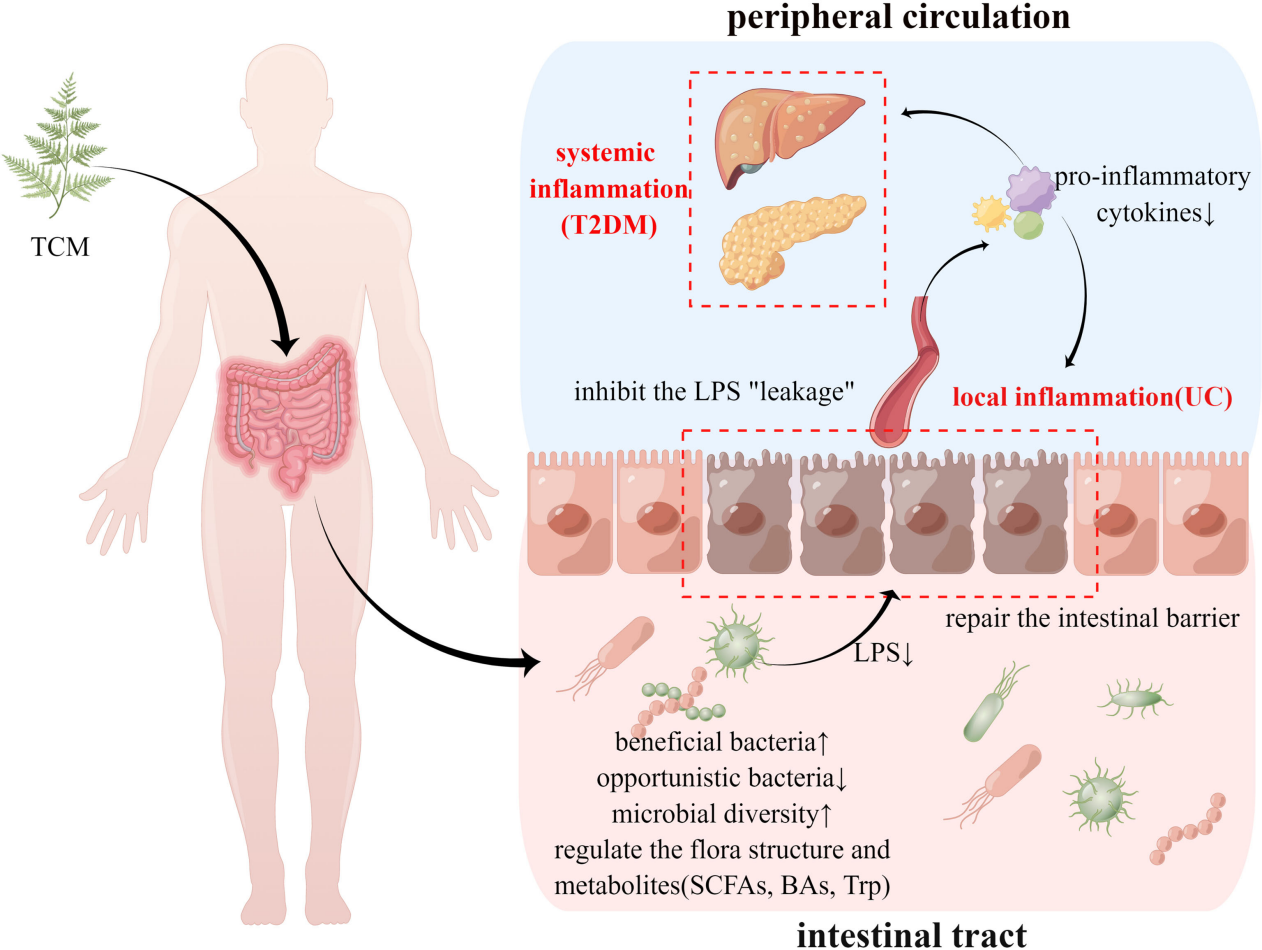
TCM’s therapeutic effect of treating UC and T2DM using the same method. TCM, traditional Chinese medicine; UC, ulcerative colitis; T2DM, type 2 diabetes mellitus; LPS, lipopolysaccharide; SCFAs, short chain fatty acids; BAs, bile acids; Trp, tryptophan.

Over 1,000 species of gut microbiota constitute a complex ecosystem that interacts with the host, and some pathological changes, such as the imbalance of flora structure, proliferation of pathogenic bacteria, and disorder of flora metabolites, may disturb the host’s homeostasis, leading to the occurrence of a variety of diseases (Geng et al., 2022). By analyzing the above articles, it is evident that the regulatory effects of TCM on the gut microbiota are mainly manifested in the following aspects (**Figure 1**).

#### 4.1.1 Regulation of the Microbiota Structure

The diversity of the gut microbiota form the basis of its physiological functions, and a reduction in diversity has been confirmed to be closely related to the occurrence of various diseases. Both clinical and basic studies have confirmed that some patients with UC and UC mouse models induced by DSS decrease gut microbiota diversity (Xu et al., 2021a; Zhuang et al., 2021), and after exclusive enteral nutrition and chemical drug treatment, α-diversity indicators show an upward trend (Radhakrishnan et al., 2022). Similarly, decreased diversity has been observed in individuals with T2DM and in high-fat-fed mouse models (Nyavor et al., 2019; Balvers et al., 2021). As described in **Tables 1**, **2**, most TCMs, such as curcumin and *Rhizoma atractylodis macrocephalae*, restore the diversity of flora, which may be one of the factors for treating different diseases using the same method. However, berberine (the main component of Rhizoma Coptidis) was confirmed to reduce bacterial diversity in both UC and T2DM models, possibly be due to its strong antibacterial efficacy (Li et al., 2021b), and its therapeutic effect on UC and T2DM may be similar to that of antibiotics. In addition, Huanglian Jiedu decoction, which uses Huanglian as the main drug, also reduced the bacterial diversity in UC mice.

Studies have demonstrated that the increased abundance of Firmicutes is associated with the development of obesity, and an upward trend in high-fat-fed animal models of T2DM has also been observed (Abenavoli et al., 2019). Some clinical studies have confirmed that the Bacteroidetes population is reduced in patients with UC, and this status could also be altered by microbiota transplantation; however, some studies have drawn contrary conclusions (Shi et al., 2016). As shown in the table, some TCMs have a clear regulatory effect on Firmicutes and Bacteroides, and most of them reduce the Firmicutes/Bacteroides ratio, which may be related to their therapeutic effect on the disease; however, considering the complexity of the gut microbiota, this conclusion requires further investigation.

#### 4.1.2 Increasing Beneficial Bacteria

*Bifidobacterium* is an important probiotic that exists in human and animal intestines and is closely related to a variety of physiological and pathological phenomena in the body. In both UC and T2DM, the abundance of *Bifidobacterium* has exhibited a downward trend (Prosberg et al., 2016; Gurung et al., 2020), and probiotics containing *Bifidobacterium* potentially play an adjuvant role in the treatment of these two diseases (Tiderencel et al., 2020; Yao et al., 2021), which may be related to the regulation of intestinal immunity, inhibition of mucosal inflammation and oxidative stress, and protection of the intestinal barrier (Hampe and Roth, 2017). As indicated in the tables, the mechanism of action of curcumin and some prescriptions, such as SLBZP and LZD, on UC may be related to the regulation of *Bifidobacterium* spp. *Akkermansia muciniphila*, which has been a research topic of interest in recent years, can increase the integrity of the intestinal barrier by activating the Toll-like receptor 2 signaling pathway and promoting the production of IL-10, increasing the level of endocannabinoids in the intestine, among other substances. (Everard et al., 2013; Naito et al., 2018) Similar to that of *Bifidobacterium*, the *Akkermansia muciniphila* population was also significantly reduced in UC and T2DM individuals, and some Chinese herbal extracts, such as berberine and Rhein, could effectively increase the abundance of this bacterium in the gut. *Lactobacillus* is also a probiotic with various effects, such as mucosal protection and promotion of intestinal peristalsis. Compared to inactive UC, the abundance of *Lactobacillus* in the intestinal tract of active UC has also shown a downward trend (Prosberg et al., 2016), and several Chinese herbal extracts potentially promote the proliferation of *Lactobacillus* in the UC model, which may also be an important factor for the efficacy of Chinese herbs. Moreover, we noticed that although some *Lactobacillus* species are also beneficial for the treatment of T2DM (Miraghajani et al., 2017), the Chinese herbs and Chinese herbal formulations listed in **Tables 1**, **2** did not increase the abundance of *Lactobacillus* in the T2DM model.

#### 4.1.3 Inhibition of Opportunistic Pathogens

Opportunistic pathogens in the gut microbiota are also important targets of TCM in the treatment of UC and T2DM. The impact of pathogenic *Escherichia-Shigella* on the host cannot be ignored; it not only affects the host through virulent factors, such as enterotoxins, adhesin fimbriae, and *Shigella*-like toxins, but also potentially damages the intestinal mucosa and affects the expression of intestinal tight junction proteins by destroying the host-cell actin cytoskeleton and stimulating the secretion of intestinal pro-inflammatory cytokines (Pawłowska and Sobieszczańska, 2017). In addition, the abundance of the opportunistic pathogens *Enterobacter* and *Enterococcus* was also negatively correlated with glucose and lipid metabolism and inflammatory indicators (Fei and Zhao, 2013; Patterson et al., 2016). TCM extracts, such as berberine and curcumin, and formula LZD potentially reduce the abundance of these bacteria, which may be the key factor for their therapeutic effect.

#### 4.1.4 Regulation of Gut Microbiota Metabolites

In addition to the gut microbiota, the relationship between gut-related metabolites and diseases has attracted extensive interest from researchers (Liu et al., 2022). Butyric acid, an important component of SCFAs, plays an important role in inhibiting intestinal inflammation and maintaining intestinal mucosal barrier integrity. The protective effects of butyric acid on the intestinal barrier are multifaceted. First, it can activate G protein-coupled receptors, which in turn activate their downstream signaling pathways and affect the differentiation and migration of intestinal immune cells. Second, butyrate can also activate PPARs; promote the expression of tight junction proteins; ensure intestinal barrier function; inhibit the activation of the NF-κB signaling pathway, that is, the expression of inflammatory factors; and promote the secretion of intestinal antimicrobial peptides (Silva et al., 2018). In addition to its effects on the gut, butyrate can directly regulate the host’s glucose and lipid metabolism, body weight, and appetite, among others, and plays an important role in maintaining the host’s metabolic homeostasis (Zhang et al., 2021a). In the intestines of patients with UC and T2DM, the content of butyric acid and abundance of butyrate-producing bacteria tend to decrease, and the intake of dietary fiber, prebiotics/probiotics, or direct supplementation with butyric acid preparations all potentially play an adjuvant-treatment role (Coppola et al., 2021; Zhang et al., 2021a). The tables show that some Chinese herbs not only directly increase the content of butyric acid in the intestine but also promote the proliferation of the following butyric acid-producing bacteria: *Prevotella*, *Faecalibacterium*, *Butyricicoccus*, and *Roseburia*, thus exerting therapeutic effects in both diseases.

Tryptophan (Trp) is also a key gut metabolite that can affect various intestinal and metabolic diseases (Agus et al., 2018). The aromatic hydrocarbon receptor (AhR) ligand, the metabolite produced by intestinal Trp-decomposing microorganisms, showed a decreasing trend in the intestines of patients with IBD (Lamas et al., 2016), and recent studies have confirmed that, as a key immunomodulator, AhR ligands potentially induce a variety of cellular and epigenetic mechanisms to attenuate inflammation (Cannon et al., 2021). Trp is also important for glucose and lipid metabolism in the host. A clinical study found that the levels of hemoglobin A1c, total cholesterol, low-density lipoprotein cholesterol, and apolipoprotein B-100 were lower in the high tertile of Trp than in the low tertile (Wang et al., 2022). Indolepropionic acid and indoleacrylic acid are substances commonly formed after Trp decomposition, both of which are beneficial for restoring the function of the intestinal epithelial barrier and reducing the inflammatory response. Another study found that higher serum IPA levels were associated with a reduced risk of developing T2DM and improved insulin secretion (Ballan and Saad, 2021). In addition, abnormalities in synthesis, metabolism, and related signal-transduction pathways of bile acids (BAs) have been observed in patients with UC and T2DM. As the cometabolite of “host and gut microbiota,” BAs potentially promote the repair of intestinal mucosa and affect the secretion of intestinal hormones (e.g., GLP-1) through takeda G protein-coupled receptor 5 (TGR 5) and farnesoid X receptor (FXR) pathways (Bromke and Krzystek-Korpacka, 2021; Xie et al., 2021). LPS molecules are vital components of the outer membrane of Gram-negative bacteria. Excessive LPS levels in the intestinal tract and circulation can trigger an immune inflammatory response and lead to the progression of UC and T2D (Usuda et al., 2021). Overall, the imbalance of the above gut-related metabolites/derivatives caused by gut microbiota disorders is a common pathological link between UC and T2D and is also a potential target of TCM’s therapeutic effects.

### 4.2 Improvement of the Intestinal Barrier and Inflammatory Response: Important Links for Herbal Medicine in Treating UC and T2DM

The intestinal barrier effectively prevents harmful substances in the intestine, such as pathogenic microorganisms, various biological macromolecules, and antigens, from entering the blood circulation. In the above studies, intestinal tight junction proteins were the most commonly used indicators for assessing intestinal damage. When stimulated by factors such as inflammation and oxidative stress, the protein function is damaged and the density of the intestinal barrier is reduced, leading to the “leakage” of inflammatory substances and LPS molecules into the blood circulation, resulting in the occurrence of chronic systemic inflammation (Monaco et al., 2021). Occludin and claudin are transmembrane proteins and ZO is a peripheral membrane scaffold protein, all of which are indispensable components of intestinal tight junctions (Vancamelbeke and Vermeire, 2017). In the studies listed in the tables, various Chinese herbs appeared to modulate the expression of these proteins and play a role in enhancing intestinal permeability.

During UC onset, the production of inflammatory cytokines, such as TNF-α and INF-γ, by lymphocytes and lamina propria immune cells in the inflammatory intestinal epithelium potentially induces the expression of apoptosis-related proteins, such as caspase-1 in the epithelial cells, while inhibiting the expression of anti-apoptotic proteins, such as Bcl-2, thus inducing apoptosis of epithelial cells. At this time, the epithelial cells adjacent to the apoptotic cells cannot effectively seal the space left by the apoptotic cells; that is, the intestinal tight junctions are damaged and intestinal mucosal permeability is increased (Xie et al., 2022a). Presumably, intestinal mucosal damage is an inevitable result of an immune inflammatory response in patients with UC, thus eventually leading to the formation of a vicious circle between the two. However, in the T2DM model, increased permeability of the intestinal barrier is an important inducer of systemic inflammation, and LPS is an important mediator of the connection between the intestine and peripheral tissues. Studies have confirmed that CD14, an important component of the LPS-receptor complex, and its related molecules, TLR-2, TLR-4, and MD-2, are expressed in human and rat islets (Vives-Pi et al., 2003), and the activation of the receptor triggers the accumulation of numerous inflammatory factors through signaling pathways, such as NF-κB, which subsequently impair the normal secretion of insulin by downregulating the expression of PDX-1 and MafA (Amyot et al., 2012). In addition, LPS can also release inflammatory factors by combining with corresponding receptors in insulin target organs, such as the liver, and fat tissue, thereby disrupting the insulin signaling pathway and insulin receptor expression, ultimately reducing insulin efficacy and leading to the occurrence of T2DM (Singer-Englar et al., 2019).

## 5 Conclusion

TCM potentially plays a therapeutic role in different diseases; however, the underlying principle has not been explained from the perspective of modern science. In this study, we comprehensively searched Chinese medicines, their extracts, and Chinese medicine compounds that can treat both UC and T2DM and found that the gut microbiota and “gut-inflammation” axis may be the key targets and important ways to achieve this pharmacological effect. In particular, the effects of most TCMs on the microbiota were related to modulating the microbiota structure, increasing the abundance of beneficial bacteria, increasing the concentration of intestinal butyrate, and inhibiting the proliferation of opportunistic pathogens. Notwithstanding, this review has the following limitations. First, most of the research conclusions listed in this review are based on animal experiments, and there is an urgent need for further evidence from human studies. Second, there is a complex interaction between the gut microbiota and host, and the causal relationship between disease improvement and the recovery of disrupted gut microbiota after intervention with TCM remains unclear. Therefore, the preliminary conclusion of TCM treating UC and T2DM by improving the gut microbiota warrants further investigation, such as the use of sterile animal models.

In fact, there are many examples of certain herbal medicines treating different diseases simultaneously. For example, *Coptis chinensis* can treat hyperglycemia, dyslipidemia, and metabolic-related fatty liver disease simultaneously, and through bidirectional regulation of gastrointestinal motility, *Astragalus* can effectively treat diarrhea and constipation. The impact of gut dysbiosis on health is multifaceted, and alterations in certain bacteria are associated with several different clinical phenotypes. Modulation of the gut microbiota potentially has therapeutic effects on a variety of diseases. The comprehensive regulatory effect of the intestinal flora on health is consistent with the “holistic view of traditional Chinese medicine”, and it also provides a new idea for research on the scientific connotation of “treating different diseases with the same method”.

## Author Contributions

Overall design and manuscript revision: LZ. Manuscript draft preparation, BZ and KL. Publication retrieval: HY, ZJ, and QD. BZ and KL contributed equally to this work. All authors have read and agreed to the published version of the manuscript.

## Funding

This research was funded by the National Key Research and Development Program of China (2019YFC1709904); the National Natural Science Foundation of China (82104835); the China Postdoctoral Science Foundation (2021M693542). The CACMS Innovation Fund (CI2021A01605).

## Conflict of Interest

The authors declare that the research was conducted in the absence of any commercial or financial relationships that could be construed as a potential conflict of interest.

## Publisher’s Note

All claims expressed in this article are solely those of the authors and do not necessarily represent those of their affiliated organizations, or those of the publisher, the editors and the reviewers. Any product that may be evaluated in this article, or claim that may be made by its manufacturer, is not guaranteed or endorsed by the publisher.
